# 

**Published:** 1885-10

**Authors:** 


					﻿^SUBSCRIPTION PRICE, $3.00 PER YEAR.
COLLABORATORS.
CHICAGO :
Professor J. A. ALLEN, M. D., LL. D.
Professor E. ANDREWS, M. D., LL. D.
Professor W. H. BYFORD, A. M., M. D.
Professor O. C. DeWOLF, A. M., M. D.
Pbofessob E. C. DUDLEY, A. B., M. D.
Professor J. H. ETHERIDGE, A. M„ M. D.
Professor C. FENGER, M. D.
Professor J. N. HYDE, A. M., M. D.
Professor E. F. INGALS, A. M., M.D.
Professor H. A. JOHNSON, M. D., LL. D.
CHARLES GILMAN SMITH, A. M„ M.D
J. F. TODD, M. D.
MILWAUKEE, WISCONSIN:
Professor N. SENN. M. D.
WASHINGTON, D. C:
S. O. RICHEY, M. D.
UNITED STATES NAVY
MEDICAL DIRECTO* J. M. BROWNE, M. D.
				

